# Circadian Rhythm and the Risk of Cardiovascular Diseases: Genetic Evidence

**DOI:** 10.14336/AD.2023.1018

**Published:** 2023-11-05

**Authors:** Lili Yan, Jun Chen, Fuhao Li, Yijie Chen, Ziwei Mei, Lei Chen

**Affiliations:** ^1^Taizhou Central Hospital (Taizhou University Hospital), Taizhou, Zhejiang, China.; ^2^Zhejiang Chinese Medical University, Hangzhou, Zhejiang, China.; ^3^Lishui municipal central hospital, Lishui, Zhejiang, China

## To the Editor,

Recent epidemiological investigations have revealed that people with blunted rest-activity circadian rhythms had a greater risk of cardiovascular disorder. Therefore, circadian rhythm dysregulation, manifested by circadian syndrome, is emerging as a novel proxy for cardiovascular dysfunction [1]. Recent experimental and clinical research suggests potential detrimental effects of circadian rhythm disturbance on cardiometabolic health, including higher BMI/obesity, blood pressure, significant dyslipidemia, inflammation, and diabetes [2]. However, the causal relationship between circadian rhythms and cardiovascular disease was unclear. Genetically speaking, circadian rhythm was referred to as proxied diurnal preference. We utilized a two-sample MR analysis model to assess the causative impact of proxied diurnal preference on five kinds of CVDs. Genetic variations linked to proxied diurnal preference derived from genome-wide association studies (GWAS) data from the UK Biobank (UKB) and 23and Me cohorts.

This study covered five kinds of CVD outcome events, including coronary atherosclerosis, myocardial infarction (MI), hypertension, atrial fibrillation (AF), and heart failure (HF). The FinnGen Biobank analysis round 5 provided the summary statistics for coronary atherosclerosis (23,363 cases and 187840 controls; https://gwas.mrcieu.ac.uk/datasets/finn-b-I9_CORATHER), MI (12801 cases and 187840 controls; https://gwas.mrcieu.ac.uk/datasets/finn-b-I9_MI/), hypertension (559 17 cases and 162837 controls; https://gwas.mrcieu.ac.uk/datasets/finn-b-I9_HYPTENS/), AF (22068 cases and 116,926 controls; https://gwas.mrcieu.ac.uk/datasets/finn-b-I9_AF/), and HF (23397 cases and 194811 controls https://gwas.mrcieu.ac.uk/datasets/finn-b-I9_HEARTFAIL_ALLCAUSE/). The random-effects IVW approach was used to carry out the primary MR analysis. We applied MR-Egger regression and weighted median methods to assess the robustness of the results due to the presence of horizontal pleiotropy in the IVW estimates. Statistical significance was defined as a two-tailed p-value of 0.05.

In this study, the IVW model as the primary MR approach revealed that genetically predicted circadian rhythm has a causal impact on the increased risk of coronary atherosclerosis (OR, 1.073; 95% CI, 1.004-1.147, P=0.038), as well as hypertension (OR, 1.072; 95% CI, 1.021-1.125, P=0.005). However, genetically predicted circadian rhythm does not significantly increase the risk of MI (OR, 1.081; 95% CI, 0.995-1.174, P=0.065), HF (OR, 1.015; 95% CI, 0.957-1.077, P=0.622), and AF (OR, 0.959; 95% CI, 0.887-1.038, P=0.302). Table 1 presents the detailed information. Cochran’s Q statistics indicated no heterogeneity evidence in the study outcome. MR Egger intercept test, an examination of the individual SNPs’ pleiotropy, showed no impact of individual SNPs' pleiotropy on this study results.

Night shift work and social jet lag have become essential factors affecting social public health problems. Repeated shifting of the endogenous circadian clock is harmful to health. Regardless of a person’s genetic susceptibility to AF, cyclical recurrent night shift work is linked to an increased incidence of genetically predicted circadian rhythm, which has a causal impact on the increased risk of coronary atherosclerosis and AF [3]. This study confirmed that genetically predicted circadian rhythm has a causal effect on the increased risk of coronary atherosclerosis. The mechanism by which circadian rhythm disturbance or dysregulation promotes atherosclerosis is related to its mediating inflammation and metabolic disorders. Although our results suggest a causal relationship between circadian rhythm and the increased risk of MI, this relationship did not reach statistical significance. In addition, this study did not discover the interactions between circadian rhythm and AF risk. Unlike this study, recent researchers reported that accelerometer-derived circadian abnormalities are significantly associated with a higher risk of AF [4].

**Table 1:** Mendelian randomization estimates circadian rhythm on cardiovascular diseases.

Phenotype	N SNPs	Methods	OR 95% CI	P value
coronary atherosclerosis	66	IVW	1.073 (1.004-1.147)	0.039
		Weighted median	1.091 (0.9901.202)	0.079
		MR Egger	1.196 (1.005-1.423)	0.048
myocardial infarction	66	IVW	1.081 (0.995-1.174)	0.065
		Weighted median	1.097 (0.968-1.243)	0.147
		MR Egger	1.185 (0.934-1.503)	0.065
Hypertension	66	IVW	1.072 (1.021-1.125)	0.005
		Weighted median	1.056 (0.977-1.143)	0.170
		MR Egger	1.097 (0.909-1.324)	0.339
atrial fibrillation	66	IVW	0.959 (0.887-1.038)	0.301
		Weighted median	0.945 (0.840-1.062)	0.344
		MR Egger	0.968 (0.769-1.219)	0.784
heart failure	66	IVW	1.015 (0.957-1.077)	0.622
		Weighted median	0.978 (0.894-1.072)	0.636
		MR Egger	1.000 (0.835-1.198)	0.998

Evidence suggests that circadian rhythm regulates the activity of the autonomic nervous system and the various cardiac ion channels, which are associated with the pathophysiologic mechanism of AF. Furthermore, a broad range of metabolic changes, such as cortisol, vascular inflammation, and oxidative stress, may partly explain the observed link between circadian disruption and AF. Recently, a study found a combined effect of night shift work and genetic risk on hypertension. They recommended that night shift work was linked to an elevated risk of hypertension, and the genetic risk changed this risk for hypertension [5]. Our MR analysis is the first study to explore the genomic relationship between circadian rhythm and CVD. Our study disclosed a substantial causal relationship between genetically predicted circadian rhythm and the increased risk of hypertension. Many factors regulate the effect of circadian rhythm on hypertension, including the brain’s suprachiasmatic nucleus, pineal gland melatonin synthesis, autonomic and central nervous, hypothalamic-pituitary-adrenal, hypothalamic-pituitary-thyroid, renin-angiotensin-aldosterone, renal hemodynamic, peptide, endothelial, vasoactive, and opioid systems constitute the key regulators and determinants of the blood pressure 24 hours profile [6]. Theoretically, medicine chronotherapy has the potential to offer these benefits. The β-adrenoreceptor blockers administration reduces adverse CV events on an overall population basis and abolishes the day/night pattern of such events. Hypertensive patients taking a long-acting lipophilic ACE inhibitor at bedtime can lessen their blood pressure spike in the morning. This medication method is effective for hypertensive patients whose blood pressure fluctuates significantly. The Hygia Chronotherapy Trial (ClinicalTrials.gov, NCT00741585) recently illustrated that hypertensive patients (n=9,552) routinely ingested one or more antihypertensive drugs at bedtime reduced the occurrence of major cardiovascular events than those with ingestion upon awaking (n=9,532) [7]. Our study added substantial evidence supporting the causal association of genetically predicted circadian rhythm with increased risk of coronary atherosclerosis and hypertension. This robust evidence may inform public health messages about night-shift work, especially its potential cardiovascular health consequences.

### Acknowledgements

This work was partially supported by grants from the General Research Project of the Education Department of Zhejiang Province (Y202248708). The funder had no role in study design, data collection and analysis, the decision to publish, or the preparation of the manuscript.
